# Spatial–Temporal Variations in NO_2_ and PM_2.5_ over the Chengdu–Chongqing Economic Zone in China during 2005–2015 Based on Satellite Remote Sensing

**DOI:** 10.3390/s18113950

**Published:** 2018-11-15

**Authors:** Kun Cai, Qiushuang Zhang, Shenshen Li, Yujing Li, Wei Ge

**Affiliations:** 1 College of Environment and Planning, Henan University, Kaifeng 475004, China; henu_caikun@126.com; 2 College of Computer and Information Engineering, Henan University, Kaifeng 475004, China; qshenu_zhang@163.com (Q.Z.); lyjlyj1122@sina.cn (Y.L.); 3 State Key Laboratory of Remote Sensing Science, Institute of Remote Sensing and Digital Earth, Chinese Academy of Sciences, Beijing 100101, China; gw7319@163.com

**Keywords:** NO_2_, PM_2.5_, Chengdu–Chongqing Economic Zone, OMI, MODIS

## Abstract

The Chengdu–Chongqing Economic Zone (CCEZ), which is located in southwestern China, is the fourth largest economic zone in China. The rapid economic development of this area has resulted in many environmental problems, including extremely high concentrations of nitrogen dioxide (NO_2_) and fine particulate matter (PM_2.5_). However, current ground observations lack spatial and temporal coverage. In this study, satellite remote sensing techniques were used to analyze the variation in NO_2_ and PM_2.5_ from 2005 to 2015 in the CCEZ. The Ozone Monitoring Instrument (OMI) and the Moderate Resolution Imaging Spectroradiometer (MODIS) aerosol optical depth (AOD) product were used to retrieve tropospheric NO_2_ vertical columns and estimate ground-level PM_2.5_ concentrations, respectively. Geographically, high NO_2_ concentrations were mainly located in the northwest of Chengdu and southeast of Chongqing. However, high PM_2.5_ concentrations were mainly located in the center areas of the basin. The seasonal average NO_2_ and PM_2.5_ concentrations were both highest in winter and lowest in summer. The seasonal average NO_2_ and PM_2.5_ were as high as 749.33 × 10^13^ molecules·cm^−2^ and 132.39 µg·m^−3^ in winter 2010, respectively. Over 11 years, the annual average NO_2_ and PM_2.5_ values in the CCEZ increased initially and then decreased, with 2011 as the inflection point. In 2007, the concentration of NO_2_ reached its lowest value since 2005, which was 230.15 × 10^13^ molecules·cm^−2^, and in 2015, the concentration of PM_2.5_ reached its lowest value since 2005, which was 26.43 µg·m^−3^. Our study demonstrates the potential use of satellite remote sensing to compensate for the lack of ground-observed data when quantitatively analyzing the spatial–temporal variations in regional air quality.

## 1. Introduction

The Chengdu–Chongqing Economic Zone (CCEZ) is the fourth largest economic region in China after the Pearl River Delta, the Yangtze River Delta, and the Beijing–Tianjin–Hebei region. The rapid economic development in the CCEZ has resulted in many environmental problems [1], including air pollution. Two important constituents of air pollution are nitrogen oxides (NOx = NO + NO_2_) and particulate matter with an aerodynamic diameter of less than 2.5 µm (PM_2.5_), both of which can inflict substantial harm to human health [2,3]. Studies have shown that high levels of NO_2_ in the air increase the risk of respiratory infections and lung damage [4]. In addition, NO_2_ constitutes one of the main factors that leads to the formation of acid rain photochemical smog and causes climate change; accordingly, NO_2_ considerably damages the atmospheric ecological environment [5]. PM_2.5_ particles are so small that they can reach the alveoli directly and penetrate into the bloodstream. Hence, long-term exposure to PM_2.5_ can lead to bronchitis and cardiovascular diseases [6]. In addition to affecting human health, PM_2.5_ is the primary cause of urban haze conditions [7].

Two main approaches—ground-based observation and satellite remote sensing observation—are employed to acquire long-term continuous observations of atmospheric NO_2_ and PM_2.5_ concentrations. However, current ground observation networks suffer from deficiencies in spatial and temporal coverage. By contrast, satellite remote sensing can be used to obtain data for most regions and therefore overcomes the insufficient spatial coverage associated with ground-based observations [8]. In recent years, many researchers have conducted numerous studies on tropospheric NO_2_ concentrations and PM_2.5_ mass concentrations using satellite data. For example, Liu et al. analyzed the spatial and temporal distributions of NO_2_ in urban agglomerations and their adjacent areas in the Pearl River Delta by using Ozone Monitoring Instrument (OMI) data; the results showed that the areas with high NO_2_ concentrations in the Pearl River Delta region were connected, that the urban group effect was significant, and that much higher concentrations were concentrated in the Pearl River Delta than in the surrounding areas [9]. Long et al. analyzed the effects of changes in tropospheric NO_2_ concentrations on human activities from 2005 to 2010 by using OMI data and found that the winter NO_2_ concentration is high and the summer NO_2_ concentration is low, while the western regions exhibit the opposite situation [10]. Krotkov et al. analyzed the changes in NO_2_ and SO_2_ concentrations in the world’s most polluted industrialized regions over the past few years (2005–2015) by using OMI data; their analysis revealed that NO_2_ and SO_2_ concentrations have declined in the past 10 years over the eastern U.S. and eastern Europe under the effects of pollution prevention and control policies, while those in China rose and then declined [11]. Guo et al. estimated the PM_2.5_ mass concentrations in eastern China using Moderate Resolution Imaging Spectroradiometer (MODIS) aerosol optical depth (AOD) product data and compared the values with monitoring data obtained from various sites (i.e., Benxi, Zhengzhou, Lushan Mountain, Guilin, and Nanning); the results showed that the locations with the most and least pollution are Zhengzhou and Nanning, respectively, which are basically consistent with ground station observations, among which the strongest correlation was found in Lushan [12].

The CCEZ was developed from the preexisting industrial base of Chengdu–Chongqing [13]. In 2007, the Chongqing Municipal Government and the Sichuan Provincial Government signed an agreement to promote Sichuan–Chongqing Cooperation and build the CCEZ together. The CCEZ was officially approved by the state council in March 2011, and the following two years witnessed substantial industrial developments. As a region with the highest urbanization level in western China, in recent years, the CCEZ has been playing an important strategic role in China’s economic and social development due to its rapid advancement of industrialization and urbanization. According to the development strategy of the CCEZ, energy is one of its leading industries. During the period between the 11th Five-Year Plan and the 12th Five-Year Plan, coal consumption was the main driving force for the development of the national economy, indicating that great challenges need to be overcome to reduce the associated nitrogen oxide and sulfide emissions [14]. Although the majority of coal-fired power plant boilers in China are currently equipped with low nitrogen combustion technology, the denitration efficiency is low [15], and the desulfurization technology is subject to resource shortages and secondary pollution in addition to the regional environment and other factors [16]. Industrial pollution and transportation pollution are the major sources of nitrogen oxides and sulfides in the CCEZ [17,18,19]. However, to the best of our knowledge, air pollution in the CCEZ region is not as widely studied compared with that in the Beijing–Tianjin–Hebei region, Yangtze River delta, and Pearl River delta in China [20,21,22]. In recent years, China has begun to pay attention to environmental monitoring. The characteristics of NO_2_ and PM_2.5_ can be measured using ground-based equipment, although the representativeness of individual sites is usually limited in a large domain. More importantly, current PM_2.5_ and NO_2_ monitoring stations in China have been established since 2013. Therefore, research on the spatial and temporal distribution and changes over long time series of pollution in the entire CCEZ is still lacking. In this paper, we used NO_2_ data from OMI and PM_2.5_ estimated by MODIS from 2005 to 2015 to statistically analyze the temporal and spatial distribution characteristics and the changes over long time series of NO2 and PM2.5 in the CCEZ as well as the main factors influencing these distributions to provide theoretical support for NO_2_ and PM_2.5_ control strategies in this area.

## 2. Materials and Methods

### 2.1. Study Area

**Figure 1:**
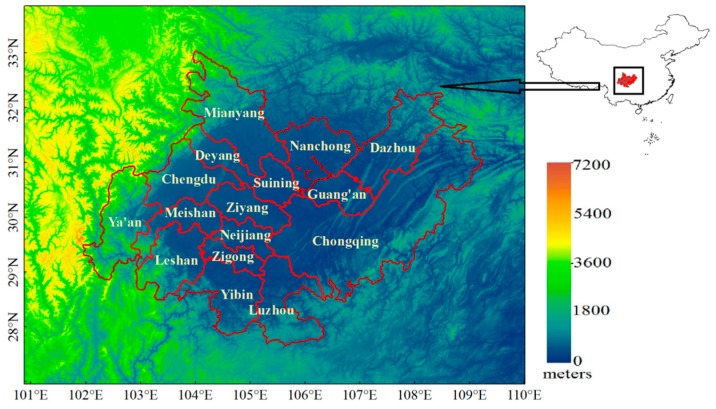
The geographic location and terrain of the study area (Chengdu–Chongqing Economic Zone) in China.

The CCEZ, which is located in the upper reaches of the Yangtze River, comprises parts of Sichuan and Chongqing, including 15 prefectures in Sichuan and 31 districts and counties in Chongqing. The entire area is located within the Sichuan Basin, which is surrounded by both high- and low-elevation areas. As shown in Figure 1, Ya’an, southern Leshan, southern Yibin, southern Luzhou, northern Mianyang, northern Dazhou, and eastern Chongqing are high-elevation areas, whereas the terrain in other areas is relatively low. The area of the CCEZ is approximately 206,000 square kilometers, and the population density is high, accounting for nearly 8% of China’s population, and its economic aggregate accounts for approximately one-third of the western region and ranks first in the west. Chengdu and Chongqing are the two central cities with the largest economic aggregate in western China.

### 2.2. OMI-Retrieved NO_2_

The OMI is one of four sensors installed on the Aura Earth Observation System satellite, which was launched by the U.S. National Aeronautics and Space Administration (NASA) on 15 July 2004. The OMI was coproduced by the Netherlands and Finland with NASA, and it is an inheritance instrument for both Global Ozone Monitoring Experiment (GOME) and Scanning Imaging Absorption Spectrometer for Atmospheric Chartography (SCIAMACHY) measurements. The orbital scanning amplitude of the OMI is 2600 km, the spatial resolution at nadir is 13 km × 24 km, and the whole world is covered once a day [23]. The OMI-retrieved NO_2_ data used in this paper were derived from the official Royal Dutch product, that is, the OMI tropospheric NO_2_ vertical column concentrations were obtained from the Derivation of OMI Tropospheric NO_2_ (DOMINO) data product version 2.0 [24] from 2005 to 2015. The product was retrieved by the Royal Institute of Meteorology in the Netherlands with a spatial resolution of 0.125°. Monthly average data were downloaded from http://www.temis.nl in an Environmental Systems Research Institute (ESRI) grid format and then converted into a .csv file format by using the Interactive Data Language (IDL); the units in this format are 10^13^ molecules·cm^−2^. Subsequently, Microsoft SQL Server was used to process the quarterly average data and yearly average data. Kayana et al. used a multiaxis differential optical absorption spectroscopy (MAX-DOAS) monitoring network in Russia and Asia to validate OMI-derived tropospheric NO_2_ vertical column concentrations. The results showed that the monthly average correlation reached an R^2^ value of 0.84 in urban areas and 0.74 in rural areas, indicating that OMI can better capture NO_2_ tropospheric levels, especially in NO_2_ source regions [25]. Mou et al. derived tropospheric NO_2_ vertical column concentrations for Hefei from ground-based MAX-DOAS observations from August 2013 to July 2014 and compared the results with OMI data; these authors reported good consistency between the data sets, with a correlation coefficient of 0.88 obtained under cloudless weather conditions [26]. Jin et al. reported that the correlation coefficient between star and ground observations in the North China Plain (Gucheng Station, Hebei, China) reached 0.945 in the absence of clouds [27]. These results show that the inversion of OMI data can reflect the spatial and temporal distributions of the tropospheric NO_2_ column.

### 2.3. MODIS-Estimated PM_2.5_

We estimated the ground-level PM_2.5_ concentrations using satellite-retrieved AOD data with a spatial resolution of 10 km in the CCEZ for 2005–2015. The AOD data were generated using the dark target algorithm based on images retrieved from the MODIS instrument onboard the Terra satellite. The processing of the MODIS AOD data product has been described at length by its development group [28].

We followed the two-stage approach developed by Ma et al. [29] to estimate the daily PM_2.5_ concentrations using satellite-retrieved AOD. The PM_2.5_ data used for model fitting were obtained from the website of the China Environmental Monitoring Center (CEMC) for all available PM_2.5_ sites located inside the CCEZ. We matched the AOD values to ground-level measurements of the PM_2.5_ concentrations (µg·m^−3^) within the same 10 km grid cell acquired on the same day that the PM_2.5_ data were collected. The first stage of the statistical model adopted a linear mixed-effect model, which was adjusted with the monitored PM_2.5_ concentrations and the same grid cell’s AOD as well as meteorological factors collected from weather stations near the grid cell. The second stage adopted a generalized additive model, which used a smoothing function to optimize the land use parameters and geographical coordinates to improve the model’s performance in predicting the PM_2.5_ concentration. This model also used meteorological parameters, such as the planetary boundary layer (PBL) height, relative humidity (RH), wind speed, and surface pressure, from the Goddard Earth Observing System Data Assimilation System (GEOS-5) as well as 300 m resolution land use data from the European Space Agency Land Cover data [30].

**Figure 2:**
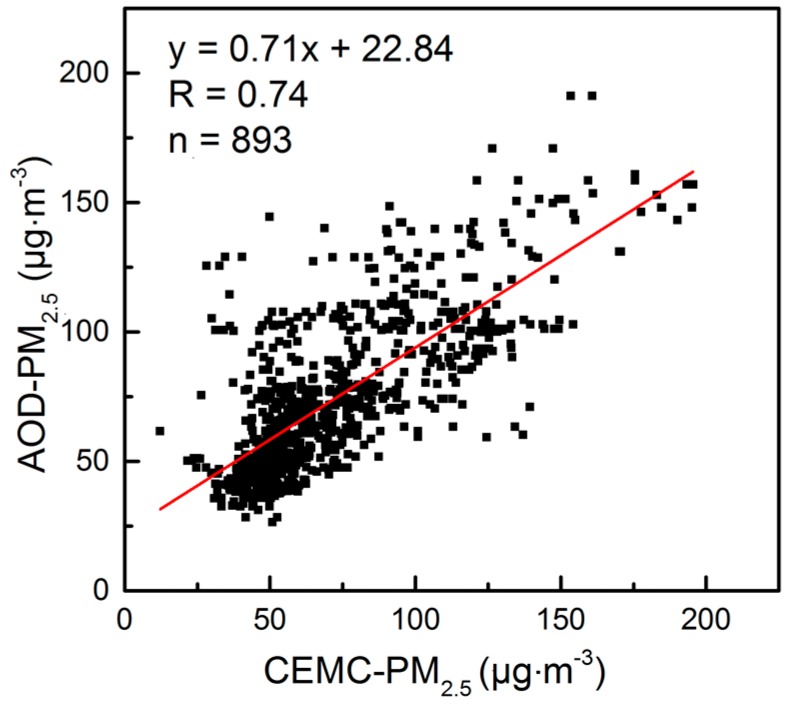
Scatter plots of the monthly PM_2.5_ (µg·m^−3^) mass concentrations over CEMC observations and AOD-estimated PM_2.5_ from 2013 to 2015.

To further illustrate the applicability of the near-surface PM_2.5_ mass concentration in the CCEZ estimated by the two-stage statistical model method, we calculated the monthly average between the PM_2.5_ mass concentrations released by the China Environmental Monitoring Center (CEMC) during 2013–2015 and estimated the PM_2.5_ mass concentrations. Until 2013, the Ministry of Environmental Protection (MEP) of China greatly expanded its air pollution monitoring network to measure hourly ground-level mass concentrations of PM_2.5_. Thus, we statistically analyzed the correlation between these concentrations, and Figure 2 shows that the correlation coefficient reached 0.74. Thus, the PM_2.5_ concentrations estimated by this method are consistent with near-surface observations. The historical data estimated by this method can be used to study the concentration level of near-surface PM_2.5_ in the study area.

## 3. Results

### 3.1. Spatial Distributions of NO_2_ and PM_2.5_ in the CCEZ

**Figure 3:**
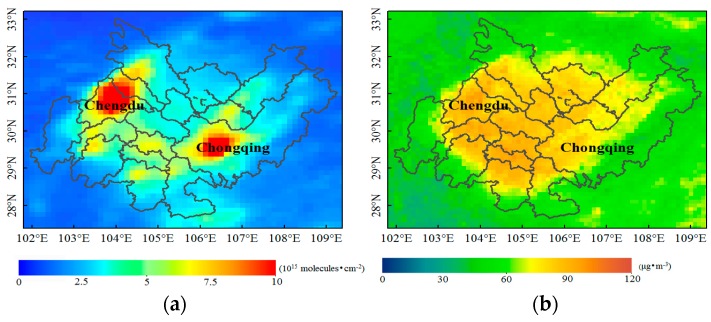
Spatial distributions of the 11-year average NO_2_ and PM_2.5_ concentrations in the CCEZ during 2005–2015: (**a**) NO_2_ columnar concentration based on the OMI DOMINO product at a resolution of 0.125° × 0.125° and (**b**) MODIS-estimated PM_2.5_ mass concentration at a resolution of 0.1° × 0.1°.

Figure 3 shows the 11-year average distributions of the tropospheric NO_2_ vertical column concentration and PM_2.5_ mass concentration in the CCEZ from 2005 to 2015. The mass concentration distribution of PM_2.5_ was similar but not identical to that of NO_2_. In addition, as shown in Figure 3b, the middle- and high-value areas of PM_2.5_ are more widely distributed than the low-value areas. The NO_2_ high-value areas (NO_2_ > 750 × 10^13^ molecules·cm^−2^) were mainly distributed in Chengdu, Deyang, and the main city of Chongqing, and the median-value (500 × 10^13^ molecules·cm^−2^ < NO_2_ < 750 × 10^13^ molecules·cm^−2^) areas were mainly distributed in Mianyang, Neijiang, and northern Yibin. According to traffic department statistics, at the end of 2014, motor vehicle ownership in Chengdu reached 3,857,000, representing a 13.9% increase from the end of the previous year; in addition, the resident population reached 13,653,000, ranking first in the province [31,32].

In Chengdu, which is located in the Sichuan Basin, the RH is very high; consequently, PM suspended in the air undergoes hygroscopic growth, thereby accelerating the accumulation of pollutants. Moreover, the weather conditions in Chengdu are fairly calm and stable, under which pollutants are not easily diluted or dispersed, leading to an increase in air pollution; additionally, inversion layers are common in Chengdu and can be relatively thick [33]. These conditions constitute important reasons why the air pollution in the CCEZ does not readily dissipate. The permanent residents in Chongqing’s main urban area account for nearly 28% of the total population in Chongqing, and they occupy an area of less than 6.7% of Chongqing. Furthermore, Chengdu and the main cities of Chongqing have a relatively well-developed traffic industry, and Deyang is a heavy industry base in China. Each of the abovementioned factors places enormous pressure on the environment, thereby resulting in serious air pollution throughout the CCEZ.

According to the 2015 Yearbook of Sichuan Province and Chongqing Municipality, the number of practitioners in the secondary industry is linearly related to air pollution. The Sichuan Province Environmental Protection Agency released a bulletin on the ambient air quality in June 2017; the ambient air quality was measured in the first half of the year, and the results regarding the magnitude of the variation in the air quality were ranked. This information showed that within the CCEZ, the cities of Sichuan Province (i.e., Chengdu, Deyang, and Neijiang) are the most polluted [34]. This result is consistent with the multiyear average mean from 2005 to 2011. The CCEZ is centered around Chengdu and Chongqing, which drive the development of the surrounding cities. According to the development of the central cities in the regional report of the CCEZ, Deyang is an important manufacturing base for heavy equipment, Zigong is a modern industrial city where machinery is manufactured, and Meishan comprises mainly locomotive manufacturing and constitutes an important transport node. The main industries of Neijiang, which is an important transport node and port city, are metallurgical building materials and automotive parts production. All of these cities exhibit serious pollution. Leshan and Ya’an form part of the clean energy industrial base; appropriately, these two cities are the least polluted areas in the CCEZ [35]. As shown in Tables S1 and S2, Ya’an has the lowest 11-year annual average NO_2_ and PM_2.5_ concentrations (i.e., 232.66 × 10^13^ molecules·cm^−2^ and 53.26 µg·m^−3^, respectively) whereas Chengdu displays the highest NO_2_ concentration (i.e., 849.46 × 10^13^ molecules·cm^−2^) and Zigong presents the highest PM_2.5_ concentration (i.e., 91.34 µg·m^−3^). The pollution in these cities is inseparable from their industrial development.

### 3.2. Monthly and Seasonal Patterns of NO_2_ and PM_2.5_ in the CCEZ

**Figure 4:**
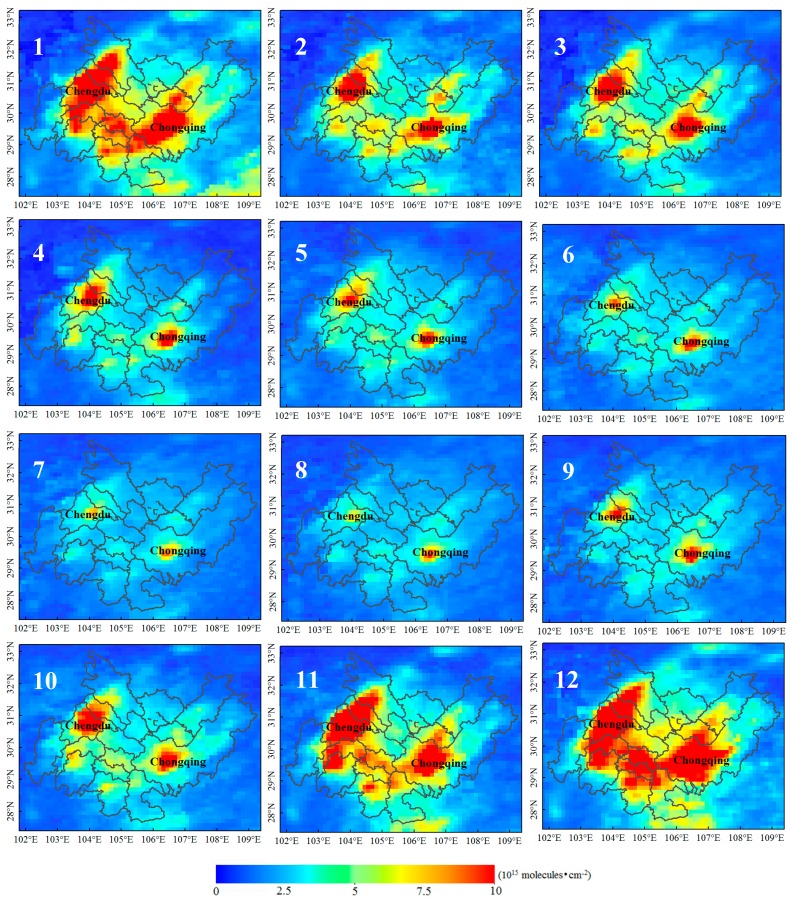
Monthly average NO_2_ distributions in the CCEZ from 2005 to 2015.

**Figure 5:**
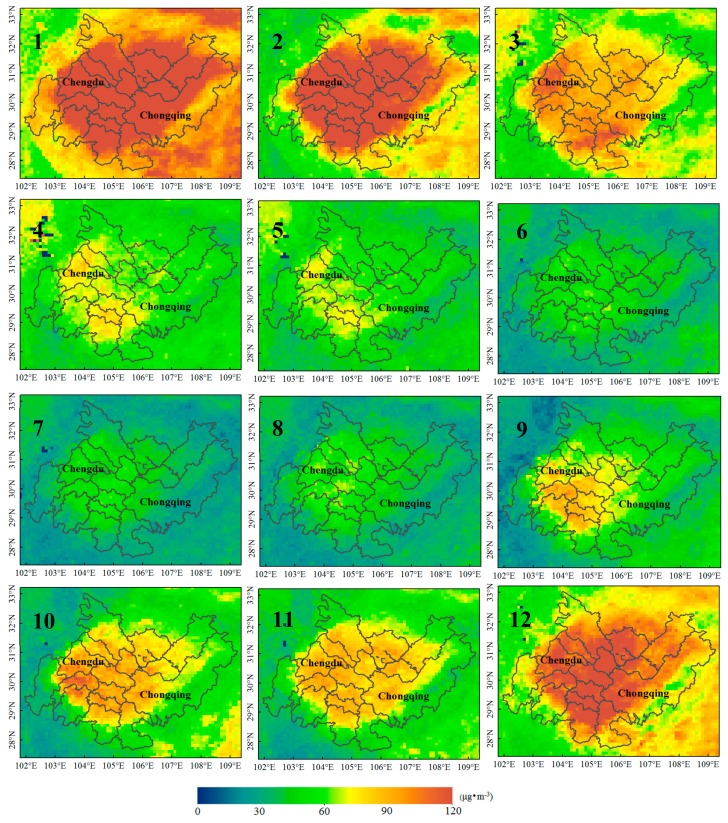
Monthly average PM_2.5_ distributions in the CCEZ from 2005 to 2015.

This paper presents the monthly average spatial distributions of the NO_2_ column concentration and PM_2.5_ mass concentration for the 11 years between 2005 and 2015. The spatial and temporal distributions of NO_2_ are shown in Figure 4. Highly polluted areas were distributed in the center of Chengdu and the main urban area of Chongqing. Along the border of the CCEZ (Ya’an, northern Mianyang, southern Leshan, northern Dazhou, and northeastern Chongqing), the pollution concentration was very low (NO_2_ < 250 × 10^13^ molecules·cm^−2^) throughout the year, and slight pollution (250 × 10^13^ molecules·cm^−2^ < NO_2_ < 500 × 10^13^ molecules·cm^−2^) was observed in December and January in southern Leshan and northeastern Chongqing. The highest levels of pollution were recorded in November, December, and January, and high-value areas were concentrated in the western and southern CCEZ and in most of the areas in and around the main urban area of Chongqing. The extent of highly polluted areas decreased considerably from February to March, and those of both highly and moderately polluted areas decreased on a monthly basis from April to August. However, the extents of areas with high and moderate pollution increased in September and October. The monthly mean distribution of PM_2.5_ is shown in Figure 5, which indicates that the overall pollution along the border area of the CCEZ was relatively low (PM_2.5_ < 60 μg·m^−3^) throughout the year. The highest levels of pollution (PM_2.5_ > 90 μg·m^−3^) were recorded in January, and even the border area pollution level, which was relatively low compared with those in other areas, was approximated high. The high-value areas in February were similar to those in January, and the pollution along the Chengdu–Chongqing boundary decreased to moderate levels. The areas with high levels of pollution decreased in March and December, and the high- and median-value areas (60 μg·m^−3^ < PM2.5 < 90 μg·m^−3^) overlapped. Higher levels of pollution were recorded in the border area in December than in February. In April and May, the extents of the high- and median-value areas decreased sharply, and areas with medium and high levels of pollution around Zigong and Chengdu intersected. From June to August, the entire area was slightly polluted; from September to November, the extents of the high- and median-value areas began to gradually expand to include most of the CCEZ, and the extents of the high-pollution areas in December were similar to but slightly heavier than those in March.

**Figure 6:**
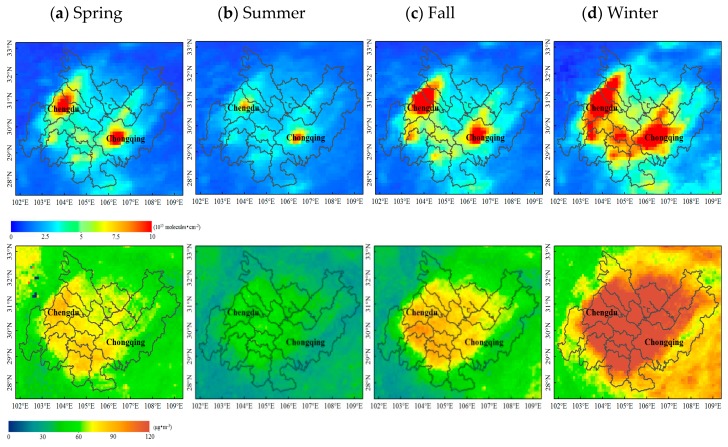
Seasonally averaged NO_2_ (**upper panel**) and PM_2.5_ (**lower panel**) concentration distributions in the CCEZ from 2005 to 2015 (Spring: March, April, and May; Summer: June, July, and August; Fall: September, October, and November; and Winter: December, January, and February).

The seasonal variations in the NO_2_ columnar concentrations and PM_2.5_ mass concentrations varied among the different areas as shown in Figure 6. The months were divided into seasons according to the climate and phenology as follows: spring comprised March, April, and May; summer comprised June, July, and August; fall comprised September, October, and November; and winter comprised December, January, and February. The NO_2_ column concentrations in Ya’an, southern Leshan, northern Mianyang, northern Dazhou, and northeastern Chongqing were very low; this result is closely related to the low elevations of these geographic locations. The NO_2_ concentrations in the center of Chengdu and in Chongqing’s downtown area were very high, and heavy pollution was most obvious during the winter. The extents of the high- and median-value areas were large, and the overall spatial and temporal distributions in the spring and fall were fairly similar. However, the extent of the high- and median-value areas in spring was obviously larger than that in fall; moreover, the lowest values were observed in summer, during which most areas were only slightly polluted. Several prominent low-value areas, namely, Ya’an, northern Mianyang, southern Leshan, and the northeastern part of Dazhou, Chongqing, were observed in terms of the PM_2.5_ concentration. The highest pollution occurred in winter; some of the borders of Chengdu and Chongqing were moderately polluted, while others were heavily polluted. The lowest pollution was observed in the summer, during which the entire CCEZ was only slightly polluted. However, differences were observed between spring and fall: the border area was more polluted in spring than in fall but the central area had relatively higher levels of pollution in fall than in spring.

**Figure 7:**
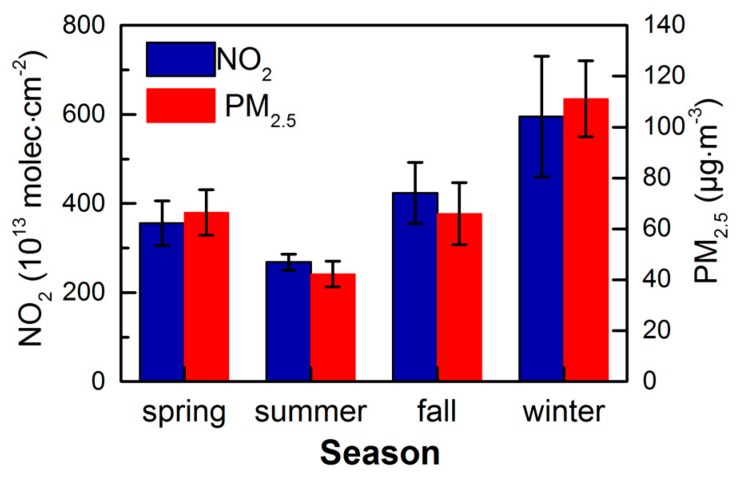
Seasonal variations in the NO_2_ and PM_2.5_ concentrations in the CCEZ from 2005 to 2015; error bars represent standard deviations.

To analyze the seasonal variation characteristics of the tropospheric NO_2_ column concentration and the PM_2.5_ mass concentration in the CCEZ, we statistically calculated the quarterly average NO_2_ column concentrations and the PM_2.5_ mass concentrations from 2005 to 2015. Figure 7 shows the quarterly average for each season spanning the 11 years from 2005 to 2015, and the error bars represent the standard deviation of the mean for a particular season over the 11-year period. The NO_2_ concentrations in the various seasons were ranked from high to low as follows: winter, fall, spring, and summer. An analysis of the sizes of the error bars in the graph indicates that the difference in the average value for a particular season over the 11-year period from 2005 to 2015 was consistent with the NO_2_ concentration. The PM_2.5_ concentration was highest in winter and lowest in summer, and it was slightly higher in spring than in fall. Within a season, the mean difference over the 11-year period was ranked as follows: winter > fall > spring > summer. In the wintertime, cold weather is dominated by downward air flow; thus, pollutants do not easily disperse, which is one of the main reasons for the higher NO_2_ and PM_2.5_ concentrations during the winter season [36]. Moreover, the CCEZ is located in the Sichuan Basin, which receives a considerable amount of rain and is consequently referred to colloquially as the “Huaxi rain screen”. In the CCEZ, dry weather occurs in winter, droughts occur in spring, floods occur during summer, and substantial rainfall occurs in fall. The annual rainfall distribution is uneven, that is, 70~75% falls from June to October, which plays a role in reducing the amount of pollution. According to research results, precipitation has little ability to remove atmospheric pollutants when the daily precipitation is less than 5 mm. However, when the daily precipitation exceeds 5 mm, the efficiency with which pollutants are removed increases with increasing precipitation. The greater the amount of daily precipitation is, the greater the clearance rate and the better the air quality. In addition, the maximum clearance rate can reach 48.55% [37,38,39]. Rainfall affects the concentration of pollutants in spring, summer, and fall, although air pollution is also affected by industrial and human factors [38].

In this study, we further processed the Multiresolution Emission Inventory for China (MEIC, http://meicmodel.org) data with a spatial resolution of 0.25° × 0.25° to estimate the anthropogenic emissions in different seasons over the CCEZ. Table 1 and Table 2 show four sources of anthropogenic NO*x* and PM_2.5_ emissions (i.e., industrial, power, residential, and transportation) in different seasons of 2012. For NO*x*, industry, power plants, and transportation were the major sources of NO*x* emissions, with industry accounting for approximately a half of the total NO*x* emissions. In addition, Table 1 clearly shows that the emissions throughout the four seasons are similar for industry, power, and transportation, while residents’ emissions are significantly higher in winter than in the other three seasons. Compared with NO*x*, anthropogenic PM_2.5_ emissions are mainly contributed by industrial and residential sources, which together accounted for more than 90% of the emissions in 2012. Similar to NO*x*, industrial emissions accounted for more than half of the total PM_2.5_ emissions. As shown in Table 2, residential sources contributed more than 64% of the PM_2.5_ emissions in winter, and these emissions are approximately three times higher than those of the other three seasons.

### 3.3. Eleven-Year Variations in the NO_2_ and PM_2.5_ Concentrations in the CCEZ

**Figure 8:**
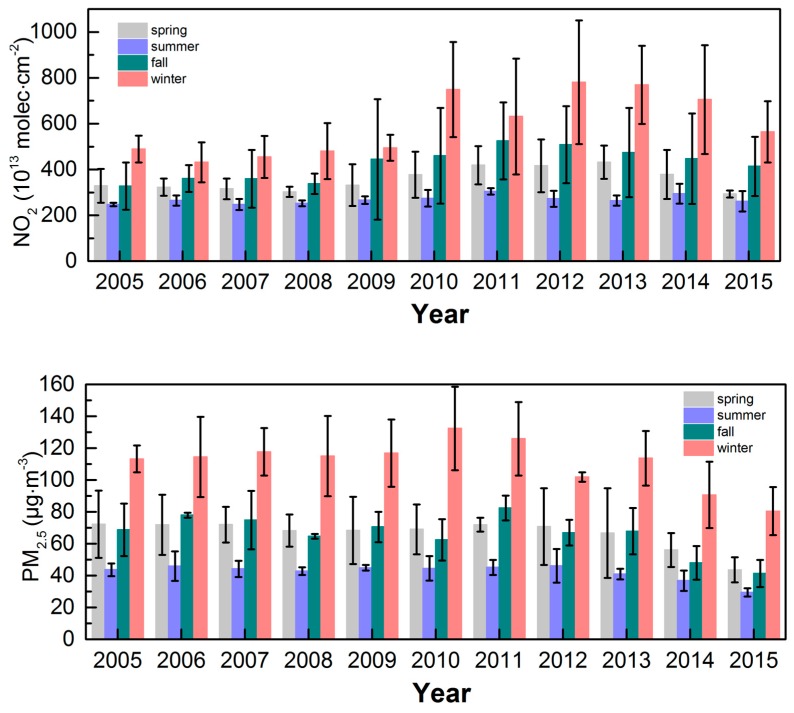
Seasonally averaged NO_2_ (10^13^ molecules·cm^−2^) (upper panel) and PM_2.5_ (µg·m^−3^) (lower panel) concentration changes in the CCEZ from 2005 to 2015; vertical bars represent standard deviations.

Figure 8 shows the quarterly average for each year of the 2005–2015 period. The error bars represent the standard deviation of the mean of all months within a season. From 2005 to 2015, the NO_2_ concentration in winter exhibited the largest fluctuation with an increase of 348.86 × 10^13^ molecules·cm^−2^ and a decrease of 184.95 × 10^13^ molecules·cm^−2^. The NO_2_ concentration increased linearly from 2006 to 2010 and reached a peak in 2010, followed by a decrease and then an increase from 2011 to 2013 and subsequently a linear decrease from 2013 to 2015. The NO_2_ concentration in spring decreased slightly from 2005 to 2008 but continued to increase from 2008 to 2013 and reached a maximum increase of 129.13 × 10^13^ molecules·cm^−2^. Over the 11-year period, the NO_2_ concentration fluctuated slightly during the summer but remained fairly stable. The NO_2_ concentration in fall increased from 2006 to 2011 and exhibited an increase of 164.32 × 10^13^ molecules·cm^−2^. After 2011, the NO_2_ concentration began to decline annually and exhibited a decrease of 111.39 × 10^13^ molecules·cm^−2^. Based on the error bars in the figure, we can conclude that the mean monthly fluctuation was typically highest in winter and then fall, spring, and summer. The fluctuations in the PM_2.5_ mass concentration during the fall and winter were relatively large with no regularity. The difference between the highest and lowest concentrations was 51.93 µg·m^−3^ in winter and 41.11 µg·m^−3^ in fall. The PM_2.5_ concentration was fairly stable in spring and summer from 2005 to 2013 but exhibited a steady decline from 2013 to 2015; this result is related to the “Suggestions on Haze Pollution in the Sichuan Basin and Suggestions for Countermeasures” formulated by the Sichuan Provincial Department of Environmental Protection in 2013 and the “Chongqing City Blue Sky” action implementation plan (2013–2017) proposed by Chongqing Municipality. The average monthly fluctuation in the PM_2.5_ mass concentration was highest in winter and lowest in summer, while the fluctuation in spring was unstable and greater than that in fall.

**Figure 9:**
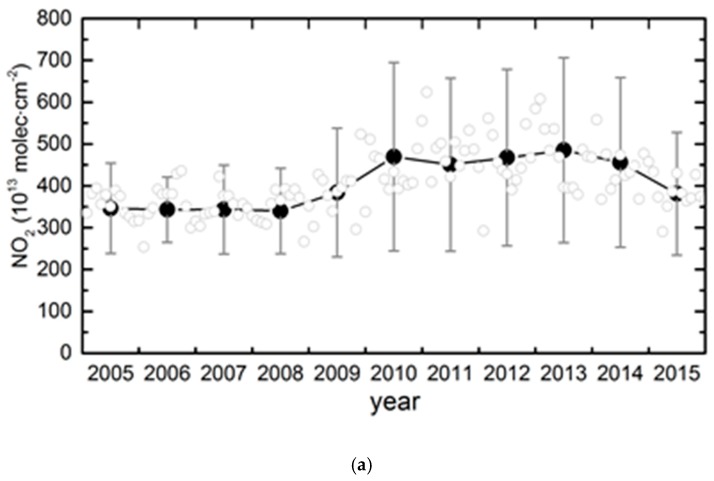

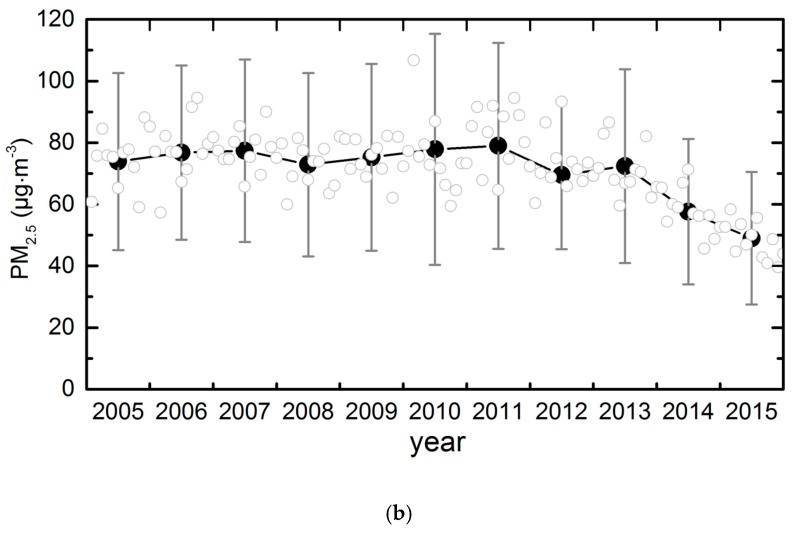
Changes in the annual average NO_2_ (**a**) and PM_2.5_ (**b**) concentrations in the CCEZ during 2005–2015; gray circles represent deseasonalized monthly columns, while black-filled circles show the annual means, and vertical bars show the standard deviations.

We used the monthly average data of 2005–2015 and the regression model developed by Lamsal et al. to compare the interannual changes over the 11-year period in the CCEZ [40,41,42]. The time series of the monthly average consists of three parts: the seasonal trend, linear trend, and irregular fluctuation:

(1) Ω(t)=S(t)+T(t)+I(t)

where t represents time (in months) [43]. In general, the seasonal trend is composed of sine and cosine functions [44]:

(2) $$S\left(t\right)=c_{0}+\overset{3}{\underset{j=1}{\sum }}\left(c_{1j}\sin\left(2\pi \mathrm{jt}/12\right)+{\;c}_{2j}\cos\left(2\pi \mathrm{jt}/12\right)\right)$$

where c_0_, c_1j_, and c_2j_ are constant coefficients. The monthly average value changes with the month; therefore, we used the 12-month moving average method to calculate the monthly index. We identified and extracted the seasonal trend components by exploiting changes in the measured seasonal pattern (amplitude and phase) for individual years. Eleven regression lines (2005–2015) are based on annual observations plus six-month observations in adjacent years. Comparing the 11 regression lines with high and low amplitudes, two seasonal terms (S(t) = s1 + s2 Equation (1)) were identified and isolated. In this paper, we calculated the changes in the deseasonalized trends of the NO_2_ columns (Figure 9a) and PM_2.5_ (Figure 9b) from 2005 to 2015. The error bars show that the monthly fluctuation in NO_2_ was greater than that in PM_2.5_. The black-filled circles in the figure show that the NO_2_ and PM_2.5_ concentrations generally exhibited upward trends from 2005 to 2011, followed by downward trends after 2013. The annual mean NO_2_ concentration was fairly similar from 2011 to 2013 but exhibited a subsequent downward trend, which was attributed to the environmental strategy formulated for the regional planning requirements of the CCEZ. The final year for the total emission reduction of major pollutants in the 12th Five-Year Plan was 2015, during which all cities in Sichuan and Chongqing Municipalities accelerated the key emission reduction projects pertaining to the total amount of major pollutants, and they achieved remarkable results. By the end of 2015, the NO_2_ concentration decreased by 15.4% compared with 2011, and the PM_2.5_ concentration decreased by 38%. In 2011, the concentrations of NO_2_ and PM_2.5_ reached their highest values of 450.71 × 10^13^ molecules·cm^−2^ and 78.96 µg·m^−1^, respectively, and in 2015, they reached their lowest values of 381.19 × 10^13^ molecules·cm^−2^ and 48.98 µg·m^−3^, respectively.

**Figure 10:**
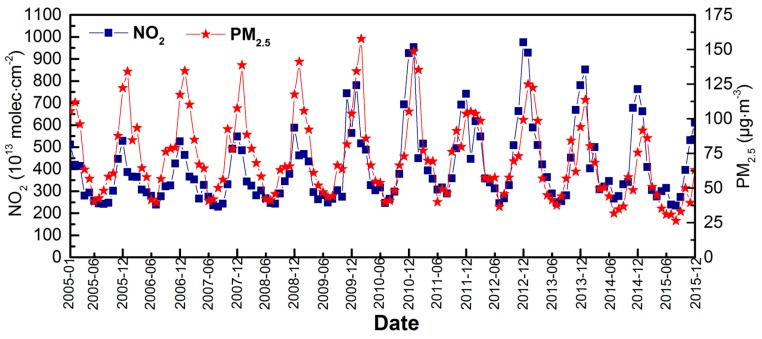
Changes in the monthly average NO_2_ (10^13^ molecules·cm^−2^) and PM_2.5_ (µg·m^−3^) concentrations in the CCEZ from 2005 to 2015.

The monthly average NO_2_ and PM_2.5_ concentrations over the CCEZ are shown in Figure 10. The monthly average concentrations of NO_2_ and PM_2.5_ showed cyclical variations throughout the year. The troughs of NO_2_ and PM_2.5_ in each cycle generally occurred in July and August (i.e., summer); NO_2_ reached a minimum value of 230.15 × 10^13^ molecules·cm^−2^ in August 2007, and PM_2.5_ reached a minimum value of 26.43 µg·m^−3^ in August 2015. The peaks usually occurred in December and January (i.e., winter); NO_2_ reached a maximum value of 975.18 molecules.cm^−2^ in December 2012, and PM_2.5_ reached a maximum of 157.64 µg·m^−3^ in February 2010. From 2005 to 2008, the tropospheric NO_2_ columnar concentration and PM_2.5_ mass concentration were fairly stable and exhibited few fluctuations. From 2008 to 2010, the NO_2_ concentration continued to increase, and the PM_2.5_ mass concentration fluctuated slightly. The concentrations of NO_2_ and PM_2.5_ dropped sharply in 2011 compared with those in previous years, and the concentrations in 2012 were unchanged from the 2010 levels. Between 2013 and 2015, the NO_2_ and PM_2.5_ concentrations continued to decrease periodically, and in August 2015, they reached the lowest values in the 11-year period.

## 4. Conclusions

This study analyzed the spatial–temporal distributions in NO_2_ and PM_2.5_ using satellite remote sensing data in the CCEZ over 11 study years. Geographically, the area with the highest level of column NO_2_ is Chengdu, which presented an average value of 848.55 × 10^13^ molecules·cm^−2^ in the 11-year study period. The area with the highest near-surface PM_2.5_ concentration is Zigong, which presented an average value of 91.34 µg·m^−3^ over the 11-year study period. The area with the lowest concentration of NO_2_ and PM_2.5_ is Ya’an, which presented average values of 91.34 µg·m^−3^ and 53.26 µg·m^−3^ over the 11-year study period, respectively.

Seasonally, the tropospheric NO_2_ column concentration and near-surface PM_2.5_ mass concentration in the CCEZ all showed obvious monthly and seasonal variation characteristics. The concentrations of NO_2_ and PM_2.5_ reach their highest annual values usually in December and January in winter and their lowest annual values in July and August in summer. For anthropogenic emissions, industrial emissions contributed approximately 50% of NOx and PM_2.5_ emissions. For PM_2.5_, residential emissions contributed more than 64% in winter, and these emissions were approximately three times higher than those in the other three seasons.

During our study period, NO_2_ presented relatively greater fluctuations. From 2005 to 2011, the concentration of column NO_2_ showed annual increases and reached a high value of 953.33 × 10^13^ molecules·cm^−2^ in January 2011, after which the column NO_2_ concentration showed a downward trend, although it increased in 2013 and then continued to decline. From 2005 to 2011, the concentration of PM_2.5_ was basically unchanged and presented an annual average of 76.52 µg·m^−3^. After 2011, the concentration of PM_2.5_ began to decline sharply, and the lowest value of 26.43 µg·m^−3^ was found in August of 2015.

## Figures and Tables

**Table 1:** Four sources of anthropogenic NO*x* emissions (i.e., industrial, power, residential, and transportation) during all seasons of 2012 in the CCEZ (Unit: 10^4^ t·yr^−1^).

Type	Spring	Summer	Fall	Winter
Industry	15.45 (49.9%)	15.59 (51.9%)	16.05 (52.2%)	15.41 (43.9%)
Power	5.17 (16.7%)	4.07 (13.5%)	4.29 (13.9%)	6.85 (19.5%)
Residential	1.49 (4.8%)	1.49 (5.0%)	1.47 (4.7%)	3.9 (11.1%)
Transportation	8.92 (28.7%)	8.91 (29.6%)	8.92 (29.2%)	8.94 (25.5%)

**Table 2:** Four sources of anthropogenic PM_2.5_ emissions (i.e., industrial, power, residential, and transportation) during all seasons in 2012 in the CCEZ (Unit: 10^4^ t·yr^−1^).

Type	Spring	Summer	Fall	Winter
Industry	9.21 (53.4%)	8.96 (53.3%)	9.2 (54.1%)	9.08 (31.7%)
Power	0.53 (3.1%)	0.41 (2.4%)	0.44 (2.6%)	0.70 (2.5%)
Residential	7.04 (40.8%)	6.99 (41.6%)	6.91 (40.7%)	18.36 (64.2%)
Transportation	0.47 (2.7%)	0.46 (2.7%)	0.47 (2.8%)	0.47 (1.6%)

## Supplementary Materials

The following are available online at http://www.mdpi.com/1424-8220/18/11/3950/s1: Table S1: The mean (plus standard deviation) NO_2_ concentrations (10^13^ molecules·cm^−2^) for the sixteen cities in the CCEZ during 2005–2015; Table S2: The mean (plus standard deviation) PM_2.5_ concentrations (µg·m^−3^) for the sixteen cities in the CCEZ during 2005–2015.
