# Supplementary material for: Spatial–Temporal Variations in NO2 and PM2.5 over the Chengdu–Chongqing Economic Zone in China during 2005–2015 Based on Satellite Remote Sensing
Source: Sensors (Basel). 2018 Nov 15;18(11):3950. doi: 10.3390/s18113950 (PMC6264014; doi:10.3390/s18113950)
Supplement: Supplementary file 1 [file sensors-18-03950-s001.pdf]

Appendix A

Table A1. The mean (plus standard deviation) NO_2 concentrations (10^{13} molecules cm^{-2}) for the sixteen cities in the CCEZ during 2005–2015.

City	2005	2006	2007	2008	2009	2010	2011	2012	2013	2014	2015	Average
Chengdu	600.44(247.49)	645.43(317.63)	694.44(342.42)	657.44(336.49)	794.60(391.19)	1028.72(665.91)	946.71(522.71)	945.06(448.31)	1139.58(694.69)	933.12(518.01)	847.40(482.81)	839.36(175.60)
Deyang	466.15(174.95)	554.15(234.99)	561.08(235.31)	548.31(200.42)	760.88(432.33)	784.98(423.31)	871.17(469.90)	737.08(332.78)	875.48(519.80)	703.93(489.88)	661.58(298.96)	684.07(137.61)
Neijiang	649.12(375.17)	481.28(109.48)	514.00(201.71)	430.25(124.61)	489.09(190.84)	617.55(299.74)	515.13(194.11)	601.12(343.49)	646.14(439.58)	546.31(287.72)	513.94(279.21)	545.81(72.56)
Meishan	421.68(187.64)	417.31(134.	428.13(162.15)	387.51(167.05)	460.30(219.34)	592.92(385.86)	569.85(383.38)	555.37(247.88)	680.62(404.17)	587.87(394.18)	549.53(241.83)	513.73(94.69)
Zigong	522.61(245.92)	432.10(129.80)	502.80(215.98)	422.49(134.00)	462.52(448.76)	551.60(284.83)	472.19(146.59)	612.26(379.81)	562.26(374.46)	503.60(296.29)	521.83(387.30)	506.02(57.05)
Guang'an	359.32(87.82)	431.59(175.05)	410.04(162.23)	426.18(223.33)	465.09(236.94)	568.74(304.88)	550.04(160.44)	531.82(239.60)	534.23(197.06)	532.99(336.67)	364.00(167.67)	470.37(76.53)
Chongqing	349.46(112.79)	369.38(99.90)	343.66(145.36)	358.49(131.70)	383.05(185.57)	467.80(218.50)	484.36(307.32)	465.80(215.60)	490.14(197.35)	490.44(277.15)	373.89(158.24)	416.04(62.35)
Yibin	421.79(240.29)	339.83(56.00)	356.41(113.38)	347.40(91.19)	335.37(273.50)	397.69(164.68)	406.19(108.34)	533.57(296.74)	453.54(263.02)	402.27(168.35)	359.48(212.17)	395.78(59.28)
Luzhou	323.95(197.14)	299.47(65.49)	307.88(85.35)	319.61(73.21)	381.58(216.05)	446.36(251.00)	396.74(172.41)	486.23(290.72)	497.35(282.91)	378.94(144.26)	349.76(195.52)	380.72(70.09)
Leshan	314.21(121.47)	313.81(72.70)	304.22(106.66)	294.68(98.91)	321.00(94.76)	465.32(243.39)	363.07(139.17)	460.44(213.39)	439.06(207.59)	415.95(219.32)	343.11(136.27)	366.80(65.94)
Suining	326.82(68.20)	320.84(98.36)	319.82(77.55)	322.29(116.35)	334.74(87.50)	407.37(206.82)	380.73(145.20)	377.48(176.99)	405.53(179.73)	426.15(150.03)	344.80(127.59)	360.60(39.95)
Ziyang	325.87(113.73)	310.17(60.70)	285.99(86.88)	316.73(119.86)	323.12(91.11)	409.20(193.98)	375.79(162.60)	371.87(216.75)	423.29(224.35)	411.46(230.87)	323.01(149.72)	352.41(47.50)
Mianyang	266.70(81.93)	275.94(79.13)	266.25(81.83)	250.22(61.13)	336.63(134.90)	383.15(219.09)	384.31(201.93)	353.53(167.41)	374.57(174.48)	341.47(129.18)	296.07(71.33)	320.80(51.06)
Nanchong	255.15(42.31)	244.62(62.61)	257.48(66.50)	245.93(74.50)	272.84(80.02)	398.79(219.96)	306.80(115.41)	303.07(116.03)	335.81(84.70)	342.57(110.46)	276.21(71.25)	294.48(48.67)
Dazhou	242.76(68.02)	248.78(35.51)	230.49(62.48)	239.40(75.86)	256.37(119.27)	309.96(155.24)	285.43(105.01)	277.16(147.32)	292.60(114.27)	305.16(111.00)	217.55(38.59)	264.15(31.44)
Ya'an	153.67(22.21)	146.45(30.46)	150.55(41.56)	194.37(40.27)	219.72(53.57)	222.92(68.17)	211.17(56.06)	258.12(73.47)	253.05(88.50)	290.12(92.25)	263.90(91.03)	214.91(49.48)

Table A2. The mean (plus standard deviation) $PM_{2.5}$ concentrations (μg m^{-3}) for the sixteen cities in the CCEZ during 2005–2015.

City	2005	2006	2007	2008	2009	2010	2011	2012	2013	2014	2015	Average
Zigong	94.13(40.30)	100.28(35.98)	100.50(39.30)	93.70(35.35)	100.25(50.19)	100.76(48.42)	104.28(39.25)	86.34(26.20)	90.10(39.61)	72.19(30.39)	63.11(31.88)	91.42(13.05)
Neijiang	89.83(32.19)	98.42(30.62)	98.60(40.02)	89.68(35.45)	94.11(37.53)	98.24(44.88)	104.63(43.92)	88.49(31.14)	92.09(40.69)	71.46(30.79)	62.25(33.87)	89.80(12.50)
Ziyang	91.22(33.49)	95.86(31.65)	95.69(35.94)	85.86(35.62)	89.73(33.68)	96.91(44.87)	98.62(39.59)	81.76(25.50)	85.33(33.47)	74.76(35.79)	57.56(27.86)	86.66(12.08)
Chengdu	88.25(36.40)	85.59(30.41)	92.95(35.74)	87.30(36.36)	86.97(35.66)	92.77(44.77)	103.34(35.08)	80.70(27.88)	93.14(40.82)	69.13(31.00)	56.70(26.38)	85.17(12.72)
Meishan	84.69(23.11)	91.52(30.56)	92.55(35.47)	88.52(35.44)	82.93(32.98)	98.35(42.39)	97.40(36.64)	82.52(23.44)	88.99(32.86)	66.90(29.76)	57.92(25.01)	84.75(12.37)
Suining	89.56(43.77)	87.52(33.65)	93.97(34.09)	87.68(41.14)	92.18(34.75)	96.70(55.71)	94.82(39.75)	79.09(33.43)	82.65(32.62)	69.41(32.14)	57.47(28.90)	84.64(12.00)
Deyang	88.87(43.02)	86.78(31.24)	86.44(28.94)	85.12(34.24)	92.81(37.41)	89.58(40.80)	98.09(33.91)	77.72(27.38)	86.74(37.30)	63.44(26.68)	53.79(24.92)	82.67(13.07)
Nanchong	85.80(44.44)	81.70(30.77)	82.60(34.85)	81.71(41.33)	90.98(46.87)	94.13(61.12)	88.37(47.69)	73.77(29.01)	76.80(35.03)	61.97(30.81)	50.59(25.01)	78.95(12.90)
Guang'an	82.56(36.02)	87.36(40.36)	84.61(38.24)	78.94(36.28)	82.45(37.13)	84.99(48.92)	85.62(39.28)	77.27(27.83)	71.05(32.15)	63.85(29.11)	53.68(25.20)	77.49(10.57)
Yibin	77.33(39.76)	84.19(35.20)	81.40(31.26)	75.03(26.16)	84.24(44.63)	78.20(38.38)	80.35(29.76)	70.48(23.34)	74.61(35.59)	60.59(25.92)	53.32(26.09)	74.52(9.75)
Leshan	67.39(19.62)	72.93(25.02)	77.81(33.80)	69.07(26.83)	70.73(26.56)	78.33(32.15)	76.81(31.24)	69.26(22.67)	72.24(29.61)	53.48(23.72)	47.13(20.74)	68.65(9.88)
Luzhou	72.03(44.54)	73.59(31.19)	73.10(29.28)	71.58(28.84)	70.34(31.44)	70.44(33.95)	71.41(30.52)	65.23(27.35)	66.67(33.42)	53.63(20.11)	48.71(21.36)	66.98(8.28)
Mianyang	67.91(23.88)	71.87(25.45)	70.50(24.89)	67.70(28.26)	74.98(27.81)	74.89(32.91)	75.47(29.61)	64.32(20.21)	68.40(27.78)	51.80(19.49)	42.58(19.53)	66.40(10.33)
Chongqing	67.33(25.57)	71.32(31.10)	71.10(32.17)	66.31(29.00)	65.30(27.67)	68.68(34.76)	67.86(32.23)	64.68(24.85)	64.75(30.91)	54.76(21.78)	47.48(19.71)	64.51(7.18)
Dazhou	67.74(30.03)	69.37(31.01)	66.82(29.63)	65.97(28.09)	68.15(27.07)	67.72(34.53)	70.23(38.99)	65.15(30.79)	64.57(30.22)	53.15(24.35)	43.60(18.37)	63.86(8.12)
Ya'an	53.55(17.05)	57.55(22.14)	59.65(21.70)	56.80(24.61)	56.46(21.04)	60.83(23.45)	60.43(17.24)	54.81(16.71)	62.85(24.44)	39.05(17.68)	33.80(14.29)	54.16(9.26)
